# CAT-Seg: cascaded medical assistive tool integrating residual attention mechanisms and Squeeze-Net for 3D MRI biventricular segmentation

**DOI:** 10.1007/s13246-023-01352-2

**Published:** 2023-11-24

**Authors:** Doaa A. Shoieb, Karma M. Fathalla, Sherin M. Youssef, Ahmed Younes

**Affiliations:** 1 Computer Engineering Department, Arab Academy for Science, Technology and Maritime Transport (AASTMT), Alexandria, 1029 Egypt; 2 Department of Mathematics and Computer Science, Faculty of Science, Alexandria University, Alexandria, Egypt

**Keywords:** Cardiovascular, Left ventricle (LV), Myocardium (Myo), Right ventricle (RV), ACDC dataset

## Abstract

Cardiac image segmentation is a critical step in the early detection of cardiovascular disease. The segmentation of the biventricular is a prerequisite for evaluating cardiac function in cardiac magnetic resonance imaging (CMRI). In this paper, a cascaded model CAT-Seg is proposed for segmentation of 3D-CMRI volumes. CAT-Seg addresses the problem of biventricular confusion with other regions and localized the region of interest (ROI) to reduce the scope of processing. A modified DeepLabv3+ variant integrating SqueezeNet (SqueezeDeepLabv3+) is proposed as a part of CAT-Seg. SqueezeDeepLabv3+ handles the different shapes of the biventricular through the different cardiac phases, as the biventricular only accounts for small portion of the volume slices. Also, CAT-Seg presents a segmentation approach that integrates attention mechanisms into 3D Residual UNet architecture (3D-ResUNet) called 3D-ARU to improve the segmentation results of the three major structures (left ventricle (LV), Myocardium (Myo), and right ventricle (RV)). The integration of the spatial attention mechanism into ResUNet handles the fuzzy edges of the three structures. The proposed model achieves promising results in training and testing with the Automatic Cardiac Diagnosis Challenge (ACDC 2017) dataset and the external validation using MyoPs. CAT-Seg demonstrates competitive performance with state-of-the-art models. On ACDC 2017, CAT-Seg is able to segment LV, Myo, and RV with an average minimum dice symmetry coefficient (DSC) performance gap of 1.165%, 4.36%, and 3.115% respectively. The average maximum improvement in terms of DSC in segmenting LV, Myo and RV is 4.395%, 6.84% and 7.315% respectively. On MyoPs external validation, CAT-Seg outperformed the state-of-the-art in segmenting LV, Myo, and RV with an average minimum performance gap of 6.13%, 5.44%, and 2.912% respectively.

## Introduction

Cardiovascular diseases (CVDs) are one of the top three causes of death globally, posing a serious threat to human health [1]. Early detection and evaluation of cardiovascular disease are critical to improving human life [1, 2]. Diagnosis of CVDs involves an extensive examination of the cardiac system [2]. In clinical practice, cardiac radiologist traces the biventricular contours during the end-systolic (ES) and end-diastolic (ED) phases, which typically requires a lot of time for skilled cardiac radiologists to analyze the MRI slices of a single patient [3]. The physiological shape of the biventricular substructures (left ventricle (LV), myocardium (Myo), and right ventricle (RV)) is affected by most cardiovascular diseases [4]. It is possible to significantly reduce the risk of developing CVDs like heart failure and ischemic heart disease by detecting biventricular morphological structure changes over an extended period of time with repetitive contouring of cardiac structure ratios or dysfunction [2]. Hence, automated biventricular segmentation has a significant impact on the detection and treatment of CVDs [3]. Moreover, the development of fast, robust, precise, and clinician-friendly segmentation tools is essential in order to increase clinician productivity and enhance patient care because the current delineation methods are very time-consuming [4].

In the era of deep learning in health care management [5, 6], classification [7, 8] and segmentation of cardiac MR images (CMRI) has drawn a lot of attention [9–22]. Various semi-automatic and automatic cardiac segmentation methods have been developed. Early segmentation methods employed semi-automatic segmentation approaches such as those presented in the work of Ding et al. [9], Sharan et al. [10] and Decourt et al. [11]. Semi-automatic methods necessitate significant user intervention, as a result, they are unsuitable for applications requiring rapid segmentation. Therefore, recent studies focused on automatic CMRI segmentation. Some are focused on LV segmentation, while others consider biventricular, performing this task in one or more stages. Lately, end-to-end deep learning segmentation models have frequently been used in conjunction with traditional methods. Table 1 summarizes the recent approaches developed to address cardiac segmentation. Some of the recent approaches lost the generalization of the model by removing patients with complex congenital intra-cardiac anatomies such as patients with univentricular hearts and patients following surgical correction of transposition of great vessels [14, 16].

**Table 1:** Previous approaches to address biventricular cardiac segmentation

Ref	First author	Dataset	Segmented structure	Method		Mean DSC
				Localization	Segmentation	
[13]	Yang et al	ACDC	Biventricular	–	Applied fuzzy attention mechanism to SegNet in both up and downsampling	0.9244
[14]	Penso et al	Two clinical datasets	Biventricular	–	Redesigned skip connection of FCN to include dense blocks, and transposed convolution was used instead of up convolution	DB1: 0.9013 DB2: 0.8920
[15]	Zhang et al	ACDC Local dataset York Sunnybrook	Biventricular	–	proposed a nested U-shape Fully Convolutional Dense Network (FCD) with compressed dense blocks	ACDC: 0.91050
[16]	Abdeltawab et al	ACDC	LV and Myo	Allocated the LV center using FCN	Applied Inception to segment LV	0.9125
[17]	Cheng et al	ACDC	Biventricular	Adopted U-Net as an initial localization stage	Applied UNet to segment the biventricular	0.9130
[18]	Dong et al	Sunnybrook	LV	–	Applied two parallel modified UNet. The endo and epi contours were obtained by averaging the two segmentation maps	0.9256
[20]	Budai et al	ACDC	Biventricular	Applied ResNet	Applied pseudo gradient calculation for the regression model to segment the biventricular	0.9133
[21]	Chen et al	Sunnybrook	LV	–	Applied ResNet as the backbone of their model to capture more discriminating information	0.9300
[19]	Wu et al	Local dataset	LV	Applied CNN to localize the center of the LV	Applied UNet to segment LV	0.9510

The majority of current segmentation models require biventricular prepositioning and redundant learning parameters, which results in poor segmentation performance. Moreover, some of the mentioned models [15, 17] don’t consider the ES phase. The difficulty of considering the ES phase is the need to handle different portions of the biventricular with varying scales. In addition, the biventricular suffers from distorted unclear borderline. To address these shortcomings, the proposed framework in this paper is inspired by ResNet and UNet of the aforementioned methods, which breaks down the segmentation process into two steps: localization and segmentation [2, 10, 14, 15, 17]. However, unlike previous methods, each step is designed with specific techniques capable of producing promising results while considering the segmentation time. An approach based on DeepLabv3+ and SqueezeNet is proposed for ROI localization. In addition, 3D-ARU architecture is proposed that combines UNet, ResNet with a spatial attention mechanism for the segmentation process. As a result, CAT-Seg, the proposed framework, can achieve efficient segmentation results, considering both the ES and ED phases in terms of DSC and Intersection over Union (IoU). The proposed deep learning framework is motivated by the depicted challenges, which impose limitations on the performance of the available cardiac segmentation frameworks. The contributions can be summarized as follows:

1. A fully automatic two-stage framework for biventricular segmentation of cardiac MRI, which eliminates the need for manual prepositioning and delineation saving cardio-radiologists time and effort. The framework surpassed the performance of cascaded detection and segmentation counterparts.
2. An enhanced version of DeepLabv3+ called SqueezeDeepLabv3+ with varying atrous rates to automatically localize the three structures of different shapes, scales, and locations within the slice, reducing learning parameters.
3. A 3D attention ResUNet architecture called 3D-ARU for cardiac segmentation. The network incorporated the attention mechanism to solve the problem of the fuzzy blurred edges of cardiac substructures.
4. A comparative analysis of the performances of established architectures in cardiac MRI segmentation with the proposed framework CAT-Seg.

## Methodology

In this section, we introduce the details of the data source used for biventricular segmentation in advance. Then, the architecture of the proposed framework for segmenting the three cardiac substructures is introduced.

### Dataset

**Fig. 1:**
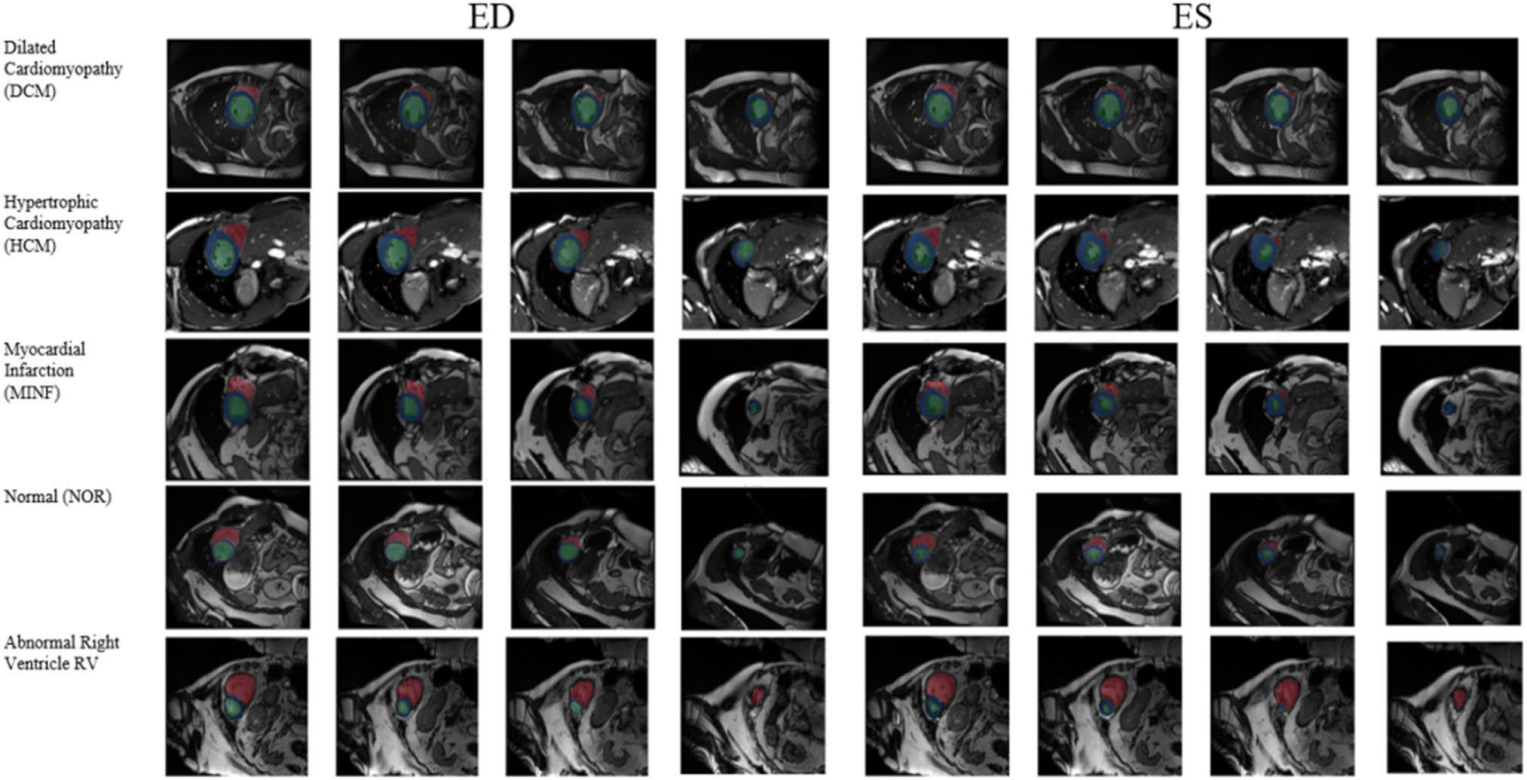
Samples from ACDC Dataset during End-diastolic and End-systolic for the four different pathologies and normal heart (LV: Green, Myo: Blue, and RV: Red)

Two datasets are used to validate the performance of our proposed framework CAT-Seg. The datasets used are the Automated Cardiac Diagnostic Challenge Dataset (ACDC 2017) [23] from the 2017 MICCAI challenge and the MyoPS dataset from the 2020 MICCAI challenge [24]. The ACDC 2017 dataset includes clinical data from 150 patients’ cardiac magnetic resonance imaging (CMRI), which included 12–35 frames of short-axis MRI in both the ED and ES cardiac phases. There were every 30 patients fell into one of the five categories: normal (NOR), dilated cardiomyopathy (DCM), hypertrophic cardiomyopathy (HCM), Myocardial infarction (MINF), and abnormal right ventricle (RV). The dataset was collected at the University Hospital of Dijon over a 6-year period using two MRI scanners with different magnetic strengths [1.5 T (Siemens Area, Siemens Medical Solutions, Germany) and 3.0 T (Siemens Trio Tim, Siemens Medical Solutions, Germany)]. The biventricular short-axis slices have thicknesses ranging from 5 to 8 mm and a spatial resolution of 1.37 1.68 mm2/pixel. Additional information about the subjects is also included in the dataset such as (ages, weights, heights, and diastolic-systolic phase instants). Samples of the dataset are depicted in Fig. 1. The biventricular contours, as previously stated, change shape and size throughout the cardiac phases. It varies according to the severity of the cardiac condition as well. The ACDC dataset provided as the training dataset consists of 100 patients and the testing dataset consists of 50 patients. For the experiments, the training dataset is randomly divided into training and validation sets. The training set consists of 80 patients, while the validation set consists of 20 patients. The test dataset consists of 50 patients.

Second, the MyoPS dataset from the 2020 MICCAI challenge is used to externally validate the performance of our proposed framework CAT-Seg without training on the dataset. It is used for external validation to investigate the robustness and the generalization performance of CAT-Seg. The MyoPS dataset includes data from 45 patients as paired three-sequence CMR images (bSSFP, LGE, and T2 CMR) and each sequence typically contains 2–6 slices. MyoPS 2020 contains 25 (102 slices) multi-sequence CMR images as a training set and 20 (72 slices) images as a testing set and it was collected using Philips Achieva 1.5T. The three CMR sequences' short-axis slices were all breath-hold, multi-slice. All patients are males suffering from myocardial infarction (MI). Three observers were used to manually label the LV, RV, and Myo from each of the three CMR sequences in order to create the ground truth segmentation. Before being employed in the creation of the ground truth segmentation, three experts in cardiac anatomy approved all of the manual segmentation results. The numerous hand delineations were averaged using a shape-based method to produce the final segmentation.

### Model

**Fig. 2:**
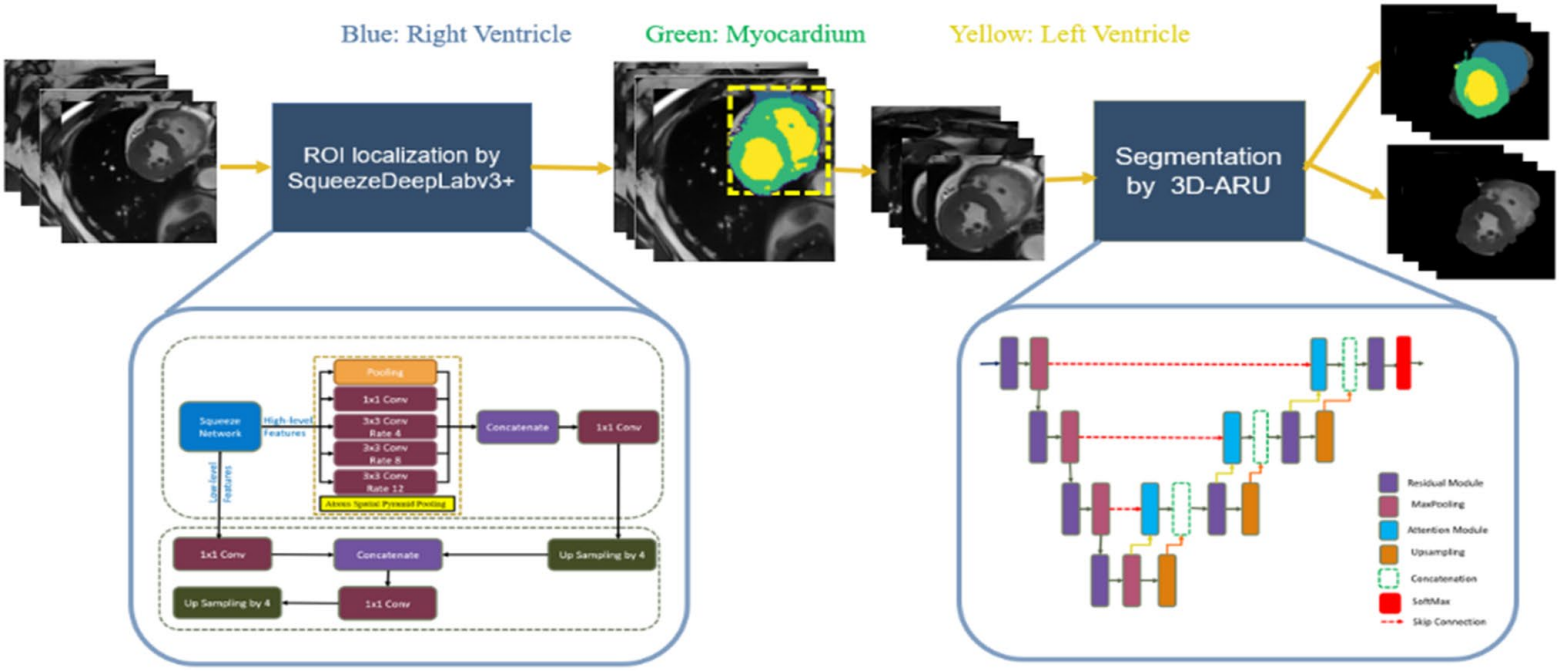
The proposed CAT-Seg framework for segmenting the biventricular system (LV, Myo, and RV)

The proposed framework consists of two stages to segment the three biventricular substructures (LV, Myo, and RV) in both cardiac phases (ED and ES). The first stage focuses on reducing the image's scope by roughly extracting the initial region of interest (ROI) using SqueezeDeepLabv3+ to overcome the problem of class imbalance as the biventricular system only accounts for a small portion of MRI slices. The second stage comprises the generation of the final LV, Myo and RV segmentations by 3D ARU and overcoming the problem of fuzzy edges due to heart movements. The details of the proposed segmentation framework are shown in Fig. 2.

#### ROI localization

For the first stage of the proposed framework, SqueezeDeepLabv3+ is proposed to extract the initial contours for LV, Myo, and RV. A relatively small region of interest (ROI) that includes LV, Myo, and RV is extracted. This step is used to reduce the scope of each volume by removing background regions that could impede the segmentation model's learning. Also, it reduces the computations performed by the proposed framework through reducing the slice size, as it focuses on the ROI only. Another advantage is the alleviation of pixel class imbalance, a prevalent issue in medical image processing [25]. In the ROI localization step, each volume is input to SqueezeDeepLabv3+, which is based on DeepLabv3+  [21] semantic segmentation network with its encoder-decoder structure. SqueezeDeepLabv3+ is used to generate masks that will be used as a guide to locate the most appropriate segments for ROI. The details of the architecture are described in more depth below.

**Fig. 3:**
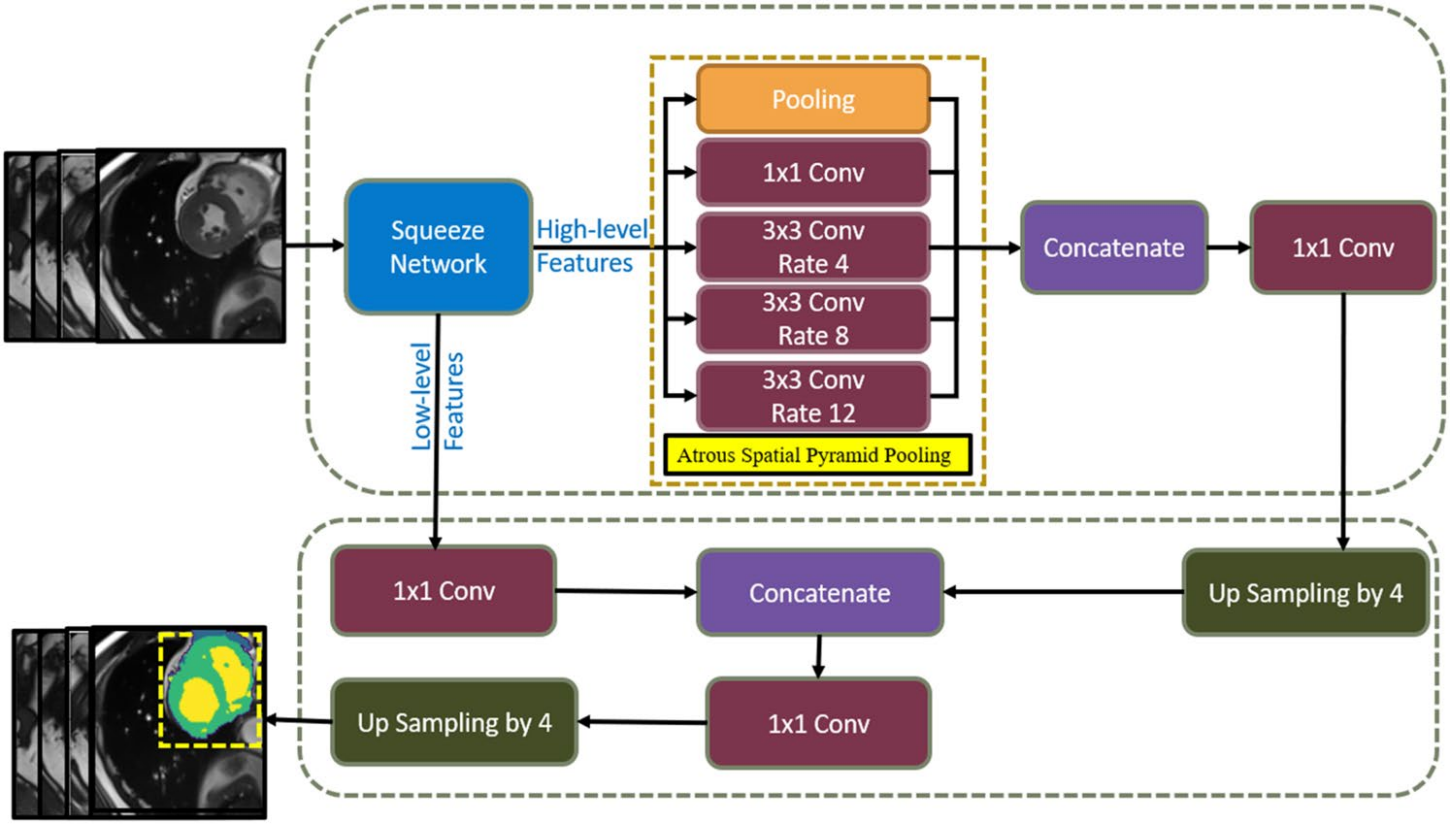
The proposed SqueezeDeepLabv3+ with SqueezeNet as backbone to enrich the network encoder. And modifying the atrous rate to localize the small objects like RV in the ES phase

SqueezeDeepLabv3+ enriches the encoder by incorporating the SqueezeNet to capture essential information from the image as shown in Fig. 3. To overcome the problem of detecting small objects with a limited number of parameters, the proposed architecture’s encoder employs a squeeze network rather than Xception in the original DeepLabv3+. Han et al. [22] proposed SqueezeNet, which is a lightweight and efficient CNN model. It has fewer parameters than Xception, and a single model’s accuracy comparable to Xception. The SqueezeNet is primarily optimized and compressed as it uses CNN microstructure optimization. It employs many 1 × 1 small convolution kernels in place of 3 × 3 convolution kernels to optimize the design of a single convolution layer, resulting in a ninefold reduction in parameters count. It also employs CNN macrostructure optimization by reducing the 3 × 3 convolution kernel's input channel count and convolution kernel parameters, splitting the convolution layer into the squeeze layer and expand layer, and encapsulating it in the fire module. The fire module is the basic unit of the SqueezeNet network that uses modular convolution. The fire module primarily consists of two layers of convolution operations, each of which connects to a ReLU activation layer: the squeeze layer which contains all 1 × 1 convolution kernels; and the expanding layer with 1 × 1 and 3 × 3 convolution kernels. The SqueezeNet model consists of nine layers of fire modules, and three levels of maximum pooling that are interspersed throughout. Furthermore, it enlarges the convolution layer perception field of vision.

The high-level semantic characteristics are then merged by an atrous spatial pyramid pooling (ASPP) module to better capture the overall semantic information of the image before the low-level features of the backbone network are fed into the decoder. The ASSP technique was inspired by the success of atrous convolutional operations and spatial pyramid pooling. (SPP) [19]. ASPP resamples feature maps produced by the encoder at various atrous rates. The results of applying a parallel convolution filter to the feature maps at various atrous rates are then concatenated in order to precisely and efficiently capture large multiscale information, as shown in Fig. 3. In this study, the ASPP module, which comprises of 1 × 1 convolution followed by 3 × 3 convolutions with different dilation rates and a max-pooling layer in parallel. The suitable dilation rates for the problem under study are determined experimentally and found to be d = 4, 8, and 12. Biventricular irregularities of different densities and sizes have been attempted to be segmented with high sensitivity using depth-wise convolution rather than standard convolution.

#### Segmentation

In the second stage, the proposed 3D-multiple attention ResUNet is used to segment the three cardiac structures (LV, Myo and RV) from the localized slices by SqueezeDeepLabv3+ . Because the LV, Myo, and RV have distinct characteristics, primarily in terms of shape and size, the ROI localization step was able to extract the area where all three structures are located. However, it occasionally failed to capture each shape, particularly in the ES cardiac cycle. To improve the segmentation process and contour each of the three structures (LV, RV and Myo), just the extracted ROI portion of the original slice will be sent to 3D-ARU in this phase.

**Fig. 4:**
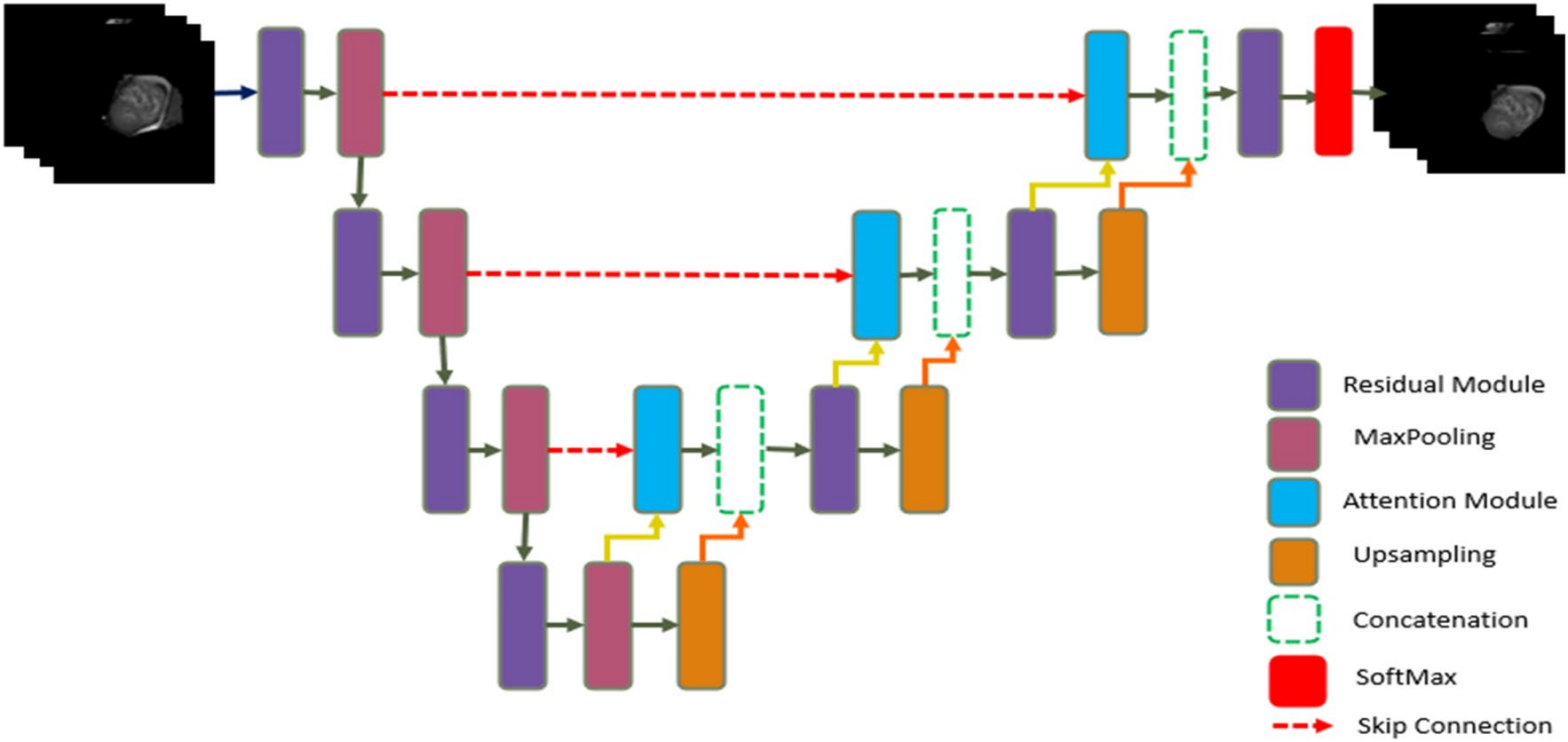
Proposed 3D-ARU model

The proposed 3D-ARU architecture, as illustrated in Fig. 4, integrates both the spatial attention mechanism and the residual module with full pre-activation. The residual module improves the channel inter-dependencies, while at the same time reducing the computational cost. It also facilitates network training. Furthermore, the rich skip connections in the ResUNet [26] contribute to the better flow of information between different layers, which enhances gradient flow during training. Due to these benefits, we use ResUNet as the foundational architecture. The encoder feature maps and the decoder feature maps are directly concatenated in the combined U-Net [30] and ResNet methods. Despite the effectiveness of ResUNet, the fuzzy boundaries in cardiac images present a challenge to the model. Therefore, the attention module is incorporated to allow focusing on the crucial regions of the feature maps.

**Fig. 5:**
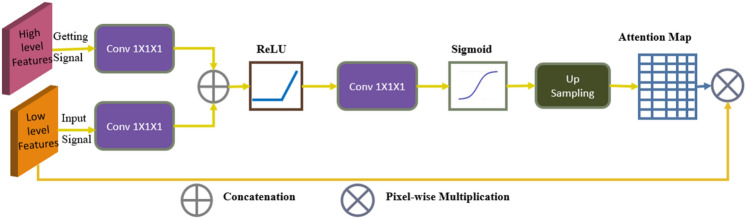
Structure of attention mechanism

We incorporated the attention block in the decoder portion of our architecture in order to be able to concentrate on the crucial regions of the feature maps, which is motivated by the success of the attention mechanism. The attention mechanism narrows its focus to a subset of its input. It focuses on a specific area of the image while ignoring the others [31] similar to human visual perception, in which they can focus on a specific point or area while suppressing the surrounding areas. By suppressing feature activations in irrelevant areas of the image, attention gates can reduce false positives [31]. In Fig. 5, the attention gate shows how the skip connection connects the encoder to the associated decoder. Two inputs are provided to the attention gate, the first of which comes from the skip connection of the associated encoder and contains all the contextual and spatial information in that layer. The second input is the gating signal from the decoder layer underneath it, and because it originates from a deeper area of the network, it has a better feature representation. It improves the learning of target regions relevant to the segmentation task while suppressing nontarget regions. First, both inputs are passed through the convolution operation and added. Following that, the first activation function, ReLU, is used, followed by the convolution operation. Furthermore, the output is resampled and passed through the second activation function Sigmoid to obtain the attention map, after which the encoder feature is multiplied pixel by pixel by the attention map to obtain the output. Figure 5 depicts a representation of the attention gate's structure.

**Fig. 6:**
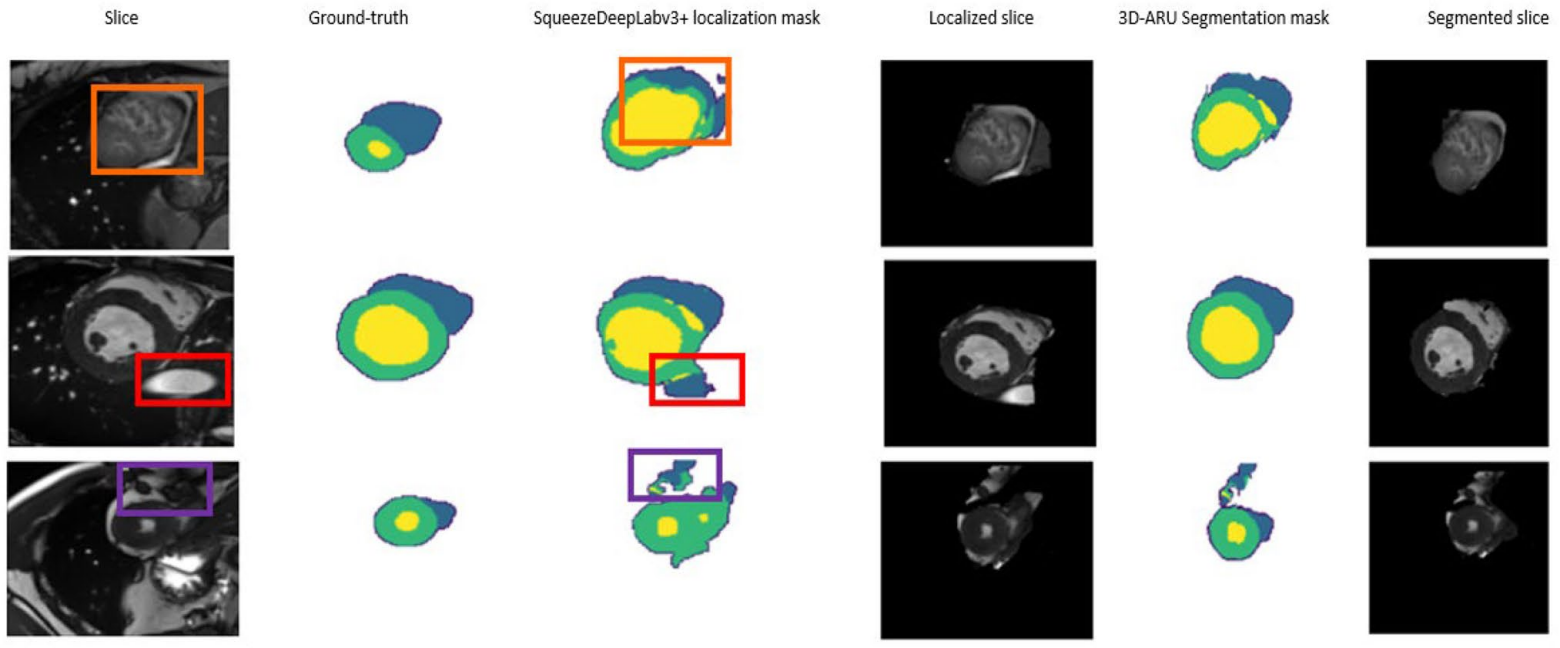
CAT-Seg final segmentation results where the RV is marked in blue, LV marked in yellow while Myocardium shown in green. Showing that segmentation results solve the problem on ROI Localized images by Removing the noisy regions that has the same intensity values as the cardiac structures, overcoming the problem of fuzzy boundaries, and extracting sharp edges. Also, it removed the overlapped tissues

Figure 6 depicts sample slices, and their ground truth together with the output of CAT-Seg. As shown in Fig. 6, the final segmentation phase identifies the contours of each of the three structures and solves the problem of fuzzy boundaries. Also, it doesn’t include other cardiac subsections as the attention module gives more attention to the boundaries and the intensities of the three structures.

### Training

Each model (SqueezeDeepLabv3+ and 3D-ARU) was trained for 100 epochs using the Adam optimizer with a learning rate of 10^–3^, a decay factor of 0.1 per epoch, and the weight decay (L2 regularization) was set to 1xe^−4^. The training set used in this case is composed of all classes of slices. The proposed 3D-ARU has 97,831,734 trainable parameters and the proposed SqueezeDeepLabv3+ has 7,051,556 trainable parameters.

### Evaluation and statistical analysis

In biventricular segmentation from MRI, the region of interest (ROI), represented by true positives (TP), is too small compared to the entire slice. True negatives represent the background. Therefore, it is necessary to focus on the Dice similarity coefficient (DSC) and intersection over union (IoU) that robustly and reliably reflect model performance [28]. The metrics used to evaluate the similarity between the proposed model’s segmentation masks and the ground truth. In this study, the performance of the proposed CAT-Seg framework was evaluated in terms of the following metrics.

The Dice similarity coefficient (DSC) is a measurement of the overlap between the foreground pixels and the ground truth foreground pixel region of the segmented image. It is the metric commonly used to gauge how effectively the medical image segmentation method works. The formula is as follows:

1 $$DSC\left(R,G\right)=2*\frac{R\cap G}{R+G}=2*\frac{TP}{TP+FP+TP+FN}$$

Another metric is the Intersection over Union (IoU), indicates the degree of dissimilarity between the segmented image's foreground pixels and the ground truth foreground pixel region. It is determined as follows:

2 $$IoU\left(R,G\right)=\frac{R\cap G}{R+G-R\cap G}=\frac{TP}{TP+FP+FN}$$

R indicates the real predicted results, and G indicates the ground truth. The true positive (TP): is the number of pixels correctly associated with the ROI, the false positive (FP): is the pixels indicated as ROI by the proposed model but as background by the ground truth, and the false negative (FN): is the pixels associated with the ROI by the ground truth but missed by the proposed model. All these values are used to determine the DSC and IoU.

## Results

In this section, the performance of the proposed architectures is verified for single-stage and multi-stage segmentation.

The performance of the proposed architectures: SqueezeDeepLabv3+ and 3D-ARU variants are tested individually as single-stage segmentation models. They are compared to available architectures depicted in Table 2. The architectures in Table 2 are chosen to present the direct counterparts of the proposed models as they can be considered as components of the proposed architectures. The obtained results are shown in Table 3. The results validate the positive effect of the proposed modification on the standard 3D-ResUNet and DeepLabv3+. As shown in Table 3, the proposed 3D-ARU improved the mean DSC of the ResUNet by 1.060, attention UNet by 2.180%, and the original UNet by 3.405%. Moreover, the proposed 3D-ARU improves the mIoU of the ResUNet by 2.050%, attention UNet by 7.080%, and the original UNet by 13.815%. In addition, the proposed SqueezeDeepLabv3+ improved the mean DSC and mIoU of the original DeepLabv3+ by 1.235% and 6.180% respectively.

**Table 2:** Model versions for the ablation experiment

Method	Description
DeepLabv3+ [27]	The original DeepLabv3+
UNet [28]	The original four-layer UNet
UNet + Attention Mechanism [29]	UNet with a spatial attention mechanism
ResUNet [26]	The original ResUNet
3D-ARU	The ResUNet incorporates with an attention mechanism
SqueezeDeepLabv3+	DeepLabv3+ with modified backbone by SqueezeNet and modified Atrous rate

**Table 3:** Results of various of single-stage experiments on the ACDC 2017 dataset

Model	Phase	DSC			Mean DSC	IoU			mIoU
		LV	Myo	RV		LV	Myo	RV	
DeepLabv3+	ED	0.9358	0.9211	0.8925	0.9164	0.8891	0.8651	0.7897	0.8479
	ES	0.9210	0.8895	0.8787	0.8967	0.7600	0.7302	0.6610	0.7170
3D-UNet [28]	ED	0.9281	0.9104	0.852	0.8968	0.7601	0.7421	0.6709	0.7243
	ES	0.9115	0.8991	0.8341	0.8815	0.7322	0.7097	0.6629	0.7016
Attention 3D-UNet [29]	ED	0.9281	0.9202	0.8859	0.9114	0.8105	0.8016	0.7628	0.7916
	ES	0.9132	0.9105	0.8506	0.8914	0.7882	0.7697	0.7492	0.7690
ResUNet	ED	0.9319	0.9268	0.8997	0.9194	0.8715	0.8536	0.8001	0.8417
	ES	0.9263	0.9122	0.8789	0.9058	0.8493	0.8404	0.769	0.8195
3D-ARU	ED	**0.9501**	**0.9342**	0.9047	**0.9296**	**0.9004**	0.8783	**0.8152**	**0.8646**
	ES	**0.9400**	**0.9202**	0.8902	**0.9168**	**0.8815**	**0.8514**	**0.7800**	**0.8376**
SqueezeDeepLabv3+	ED	0.9425	0.9213	**0.9192**	0.9276	0.8919	**0.8801**	0.8123	0.8614
	ES	0.9317	0.9023	**0.8966**	0.9102	0.8601	0.8421	0.7791	0.8271

**Fig. 7:**
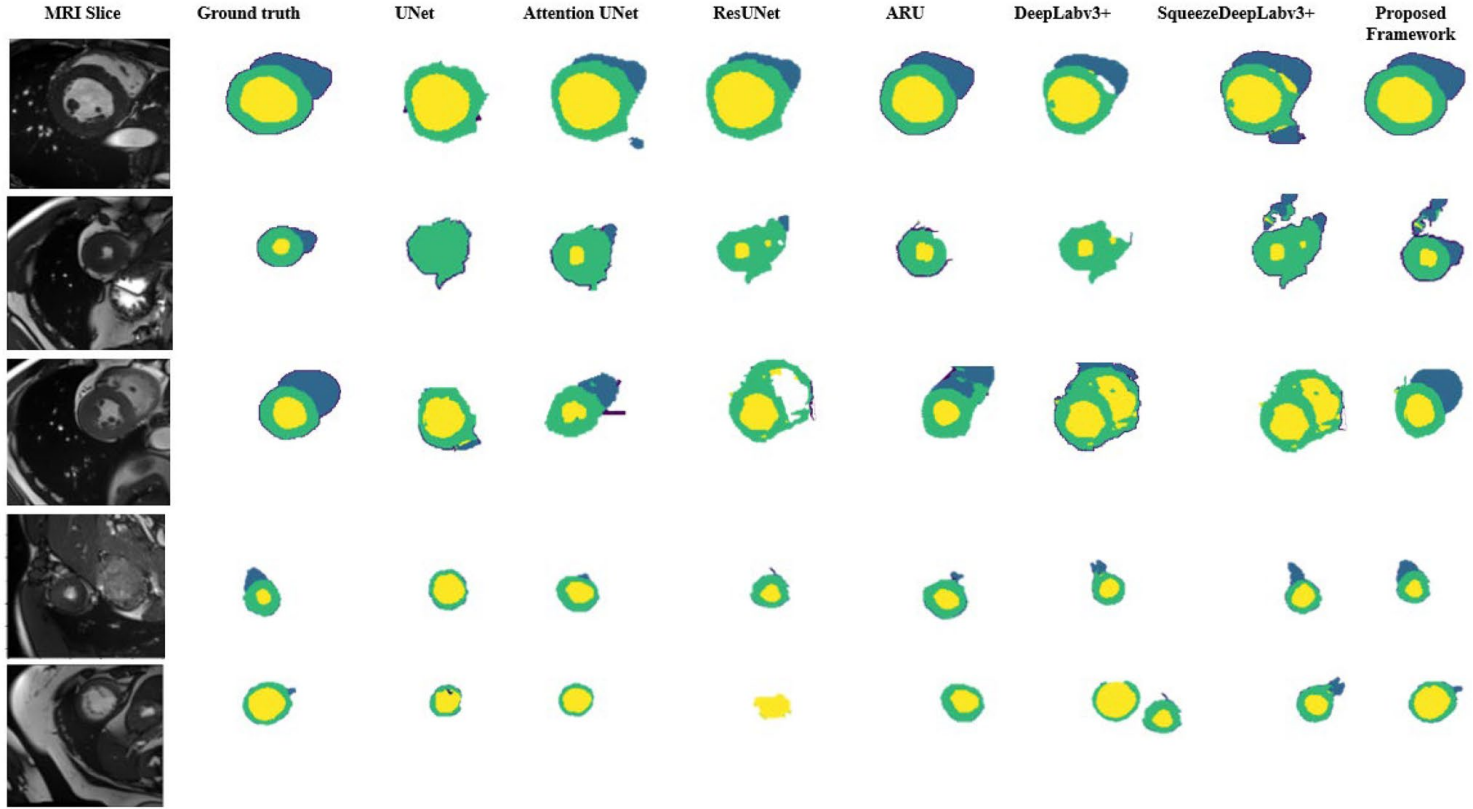
The effect of the 3D-ARU model in terms of fitting the shape of the (LV in yellow, Myo in green, and RV in blue) cardiac substructures. From left to right, the images are the original cardiac MRI slice, the ground truth, the UNet model segmentation result, attention UNet and the attention ResUNet (3D-ARU) segmentation result on ACDC dataset

Figure 7 depicts sample segmentation results of existing architecture and the two proposed variants SqueezeDeeplabv3+ and 3D-ARU to allow visual inspection. The ground truth shows that the thickness of the myocardium wall is uneven, and the edge contour of the biventricular is fuzzy and difficult to extract along with irregularity in the biventricular shape. With the use of an attention mechanism, the proposed 3D-ARU model is able to extract the edge information effectively, and the reconstructed LV and Myo contours were significantly better than those of the UNet, attention UNet, and ResUNet models. It demonstrates that the incorporation of the attention mechanism solves the problem of the fuzzy edges but still the problem of segmenting the small object such as RV persists. In the lower bottom row, the role of the modified SqueezeDeepLabv3+ with different atrous rates is elucidated in detecting small objects such as RV. DeepLabv3+ misses segmenting some tissues as Myo and LV due to its larger atrous rate. Moreover, ResUNet was unable to segment Myo and RV due to fuzzy boundaries. In addition, UNet was able to segment Myo and LV but with an enlargement of LV and thinner Myo contour. ARU solve some of the UNet, attention UNet and ResUNet such as fuzzy boundaries but failed to extract the RV. Hence, it can be seen ARU and SquzzeDeepLab3+ complement the functionality of each other so a two-stage segmentation model would be expected to yield better results. CAT-Seg output is shown in the proposed framework column, which depicts the favorable effect of their combination.

In the following, the effectiveness of CAT-Seg is experimentally verified against various two-level segmentation. The ROI localization is performed by either 3D-ARU or SqueezeDeepLabv3+, followed by fine segmentation. The localized ROIs are input to four architectures namely: 3D-UNet, Attention 3D-UNet, 3D-ARU, and SqueezeDeepLabv3+ for segmentation. 3D-UNet, and Attention 3D-UNet are selected for the coming experiment as they are frequently used in similar studies [14, 17–20, 30, 31]. All sets comprise the volumes of the same patients.

Table 4 presents the segmentation results (DSC and IoU) of the different combinations for multistage ROI extraction and segmentation. First, 3D object detector frameworks namely Mask R-CNN [27], and Retina U-Net [28] have been deployed to automatically detect a bounding box encompassing the heart in CMRI. The detected bounding box is then used for cropping the full images. Object detection performance is the contrasted to multigrain segmentation. Mask R-CNN is an extension of the Faster R-CNN [29] architecture that adds a branch for predicting object masks in parallel with the existing branch for bounding box recognition. This allows it to provide more precise object localization and instance segmentation. Retina U-Net 3D is a 3D extension of the RetinaNet architecture that is designed for volumetric medical image analysis. It uses a U-Net-like architecture with a feature pyramid network to detect 3D objects in medical images. CAT-Seg outperforms the usage of Mask R-CNN as a 3D detection framework instead of SqueezeDeepLabv3+ in segmenting LV, and Myo by 0.8909%, and 0.3526% respectively. Also, it outperforms the combination of using Mask R-CNN with SqueezeDeepLabv3+ in segmenting LV, Myo, and RV 0.9775%, 0.8515 and 0.558% respectively. Despite the usage of Mask R-CNN instead of SqueezeDeepLabv3+ in segmenting RV outperforms the CAT-Seg framework by 0.0528%, it increases testing time by 0.4210%. Moreover, the CAT-Seg framework outperforms the combination of using Retina U-Net with 3D-ARU in segmenting all the substructures. Also, for localization, the cascading of two consecutive 3D-ARU presents higher DSC in cases of segmenting Myo and RV in ES phase. However, the differences when compared to CAT-Seg is limited to 0.24% and 0.04% in case of Myo and RV respectively. In addition, the cascaded 3D-ARU testing time is 2.4 × higher than the proposed CAT-Seg. In addition, the testing time of using 3D-ARU as localization and then segmenting by squeezeDeepLabv3+ is 1.2368 × higher than the CAT-Seg. The CAT-Seg outperforms the cascaded SqeezeDeepLabv3+ by 0.11% and 0.46% in terms of mean DSC and mIoU respectively. The proposed CAT-Seg presents a performance gap of 4.87% and 15.78% compared to using 3D-ARU in localization and UNet in segmentation in terms of mean DSC and mIoU respectively. Although the combination of using SqueezeDeepLabv3+ for localization and UNet for segmentation has the lowest testing time, CAT-Seg outperforms it by 4.88% and 15.8% in terms of mean DSC and mIoU respectively. Moreover, CAT-Seg, approximately, has testing time as the combination of squeezeDeeppLabv3+ and attention UNet but CAT-Seg draws a performance gap of 4.29% and 9.66% in terms of mean DSC and mIoU respectively. While the testing time of the cascaded squeezeDeepLabv3+ is 0.9210 × the testing time of the CAT-Seg, the mean DSC and the mIoU of the CAT-Seg are 3.29% and 2.22% better than the cascaded squeezeDeepLabv3+. Therefore, CAT-Seg is elected as the proposed model rather than any other cascaded approach.

**Table 4:** Evaluation of the different experimental scenarios in terms of DSC, IoU and testing time by extracting ROI by applying 3D Detection frameworks (Mask R-CNN and Retina U-Net) ROI and the two proposed models in segmentation. Extracting ROI by applying each of the proposed models at the localization phase and applying all four models (UNet, attention Unet, 3D-ARU, and SqueezeDeepLabv3+) in segmentation

Model			Cardiac phase	DSC			Mean DSC	IoU			mIoU	Testing Time in mins
ROI extraction		Segmentation		LV	Myo	RV		LV	Myo	RV		
Detection	Mask R-CNN	3D-ARU	ED	0.9632	0.9500	0.9516	0.9514	0.9307	0.8895	**0.8542**	0.8783	5.4
			ES	0.9614	0.9407	**0.9417**		0.9105	0.8721	**0.8130**		
		SqueezeDeepLabv3+	ED	0.9627	0.9500	0.9508	0.9512	0.9332	0.8846	0.8481	0.8754	4.4
			ES	0.9604	0.9407	0.9310		0.9125	0.8621	0.8121		
	Retina U-Net	3D-ARU	ED	0.9683	0.9501	0.9402	0.9495	0.9281	0.8908	0.8435	0.8764	4.4
			ES	0.9577	0.9442	0.9369		0.9099	0.8746	0.8116		
		SqueezeDeepLabv3+	ED	0.9613	0.9486	0.9401	0.9458	0.9372	0.8918	0.8469	0.8795	4.1
			ES	0.9541	0.9394	0.9316		0.9107	0.8706	0.8198		
Localization	3D-ARU	3D-UNet	ED	0.9390	0.9348	0.8805	0.9081	0.7882	0.7591	0.6805	0.7267	4.7
			ES	0.9205	0.9302	0.8441		0.7485	0.7181	0.6662		
		Attention 3D-UNet	ED	0.9481	0.9210	0.9051	0.9142	0.8264	0.8024	0.7792	0.7881	5.1
			ES	0.9298	0.9184	0.8632		0.7990	0.7719	0.7501		
		3D-ARU	ED	0.9729	0.9534	0.9519	**0.9573**	**0.9421**	0.9002	0.8454	0.8817	8.5
			ES	0.9696	**0.9552**	0.9412		0.9191	0.8814	0.8106		
		SqueezeDeepLabv3+	ED	0.9713	0.9521	**0.9546**	0.9557	0.9357	0.9000	0.8490	0.8799	4.7
			ES	**0.9751**	0.9478	0.9337		0.9115	0.8811	0.8025		
	SqueezeDeepLabv3+	3D-UNet	ED	0.9382	0.9360	0.8790	0.9080	0.7875	0.7594	0.6800	0.7265	**3.1**
			ES	0. 9201	0.9312	0.8439		0.7477	0.7186	0.6660		
		Attention 3D-UNet	ED	0.9472	0.9210	0.9052	0.9139	0.8244	0.8024	0.7793	0.7879	3.7
			ES	0.9284	0.9188	0.8633		0.7987	0.7720	0.7509		
		SqueezeDeepLabv3+	ED	0.9513	0.9221	0.9138	0.9239	0.9057	0.8871	0.8206	0.8623	3.5
			ES	0.9451	0.9107	0.9009		0.8925	0.8780	0.7904		
		3D-ARU: CAT-Seg	ED	**0.9732**	**0.9540**	0.9515	0.9568	0.9418	**0.9006**	0.8510	**0.8845**	3.8
			ES	0.9687	0.9528	0.9408		**0.9196**	**0.8815**	0.8127		

To emphasis the highest Dice, IoU, Mean Dice and MIoU for each combination of differnt localization and sgementation models

**Fig. 8:**
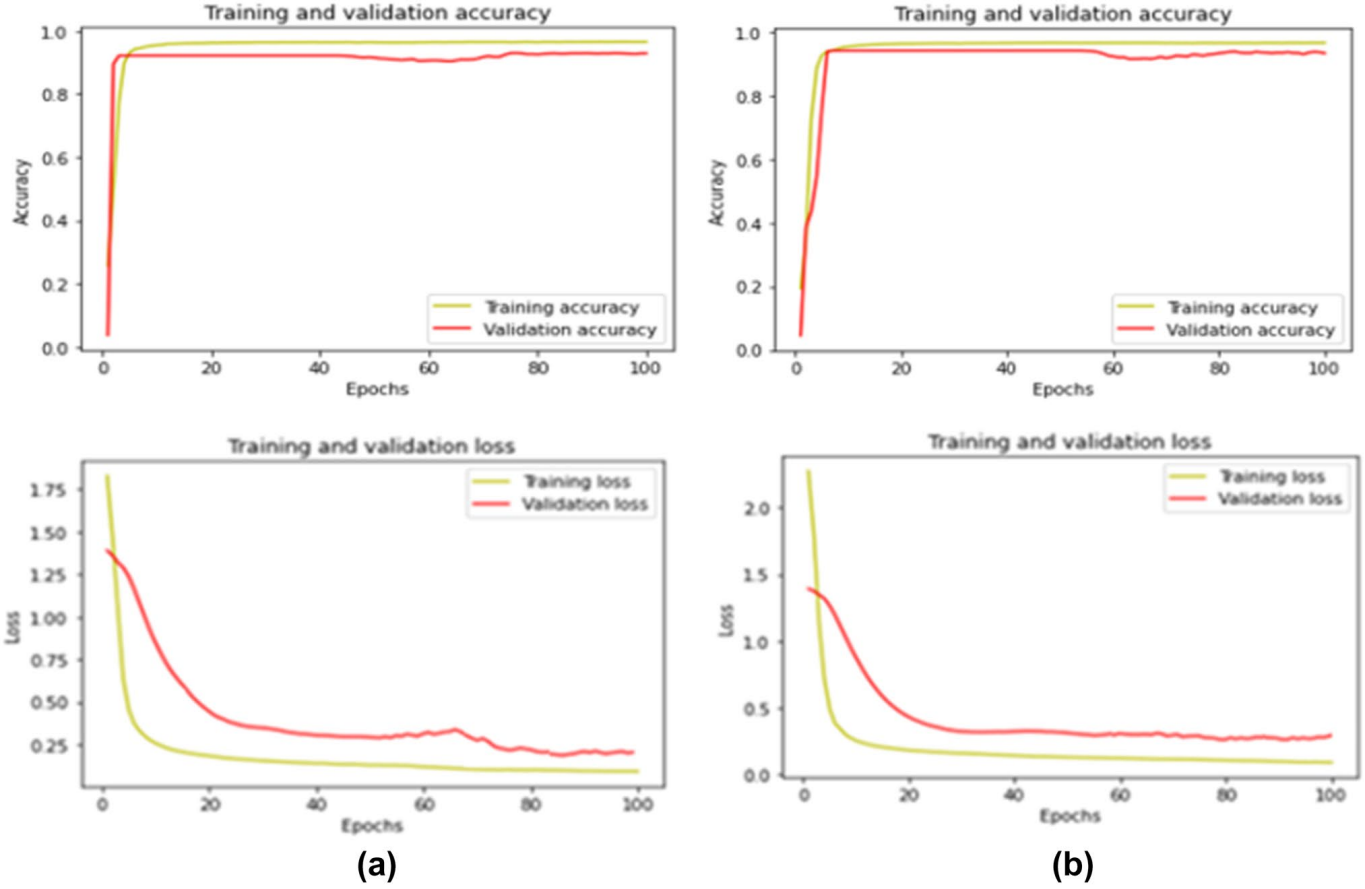
CAT-Seg DSC accuracy and loss during the training and validation process of segmenting cardiac biventricular during both cardiac phases ED in **a** and ES in **b**

Figure 8 shows the training and validation learning curves for both cardiac phases (ES and ED) using CAT-Seg. It demonstrates that both cardiac cycles have a similar trend in the training and validation stage with small performance gap diminishing the possibility of overfitting.

In addition, to make full use of the limited training data and show the performance stability and robustness, the training and testing set has been combined to apply fivefold cross-validation where each fold consists of 30 patients such as 6 patients from each pathology. The experimental results show that the DSC and IoU of the segmentation results of the biventricular regions on the test set increase significantly by using cross-validation for both stages of the CAT-Seg framework and the overall pipeline. Table 5 illustrates the improvement in each of the cardiac structures when fivefold cross-validation has been applied.

**Table 5:** Evaluation of the CAT-Seg Framework and each stage separately in terms of DSC, IoU for fivefold cross-validation on ACDC dataset

Model	Phase	DSC			IoU		
		LV	Myo	RV	LV	Myo	RV
3D-ARU	ED	0.9539 ± 0.0057	0.9400 ± 0.0061	0.9112 ± 0.0204	0.9121 ± 0.0085	0.8806 ± 0.0098	0.8213 ± 0.0209
	ES	0.9508 ± 0.0081	0.9279 ± 0.00674	0.8986 ± 0.0218	0.8903 ± 0.0099	0.8589 ± 0.0110	0.7915 ± 0.0218
SqueezeDeepLabv3+	ED	0.9467 ± 0.0066	0.9241 ± 0.0075	0.9201 ± 0.0092	0.9002 ± 0.0101	0.8896 ± 0.0108	0.8214 ± 0.0175
	ES	0.9381 ± 0.0084	0.9023 ± 0.0081	0.9063 ± 0.0140	0.8698 ± 0.0109	0.8517 ± 0.0112	0.7901 ± 0.0183
CAT-Seg	ED	0.9808 ± 0.0041	0.9602 ± 0.0045	0.9590 ± 0.0089	0.9489 ± 0.0056	0.9101 ± 0.0071	0.8604 ± 0.0104
	ES	0.9707 ± 0.0045	0.9588 ± 0.0058	0.9466 ± 0.0097	0.9204 ± 0.0078	0.8899 ± 0.0073	0.8211 ± 0.0110

**Fig. 9:**
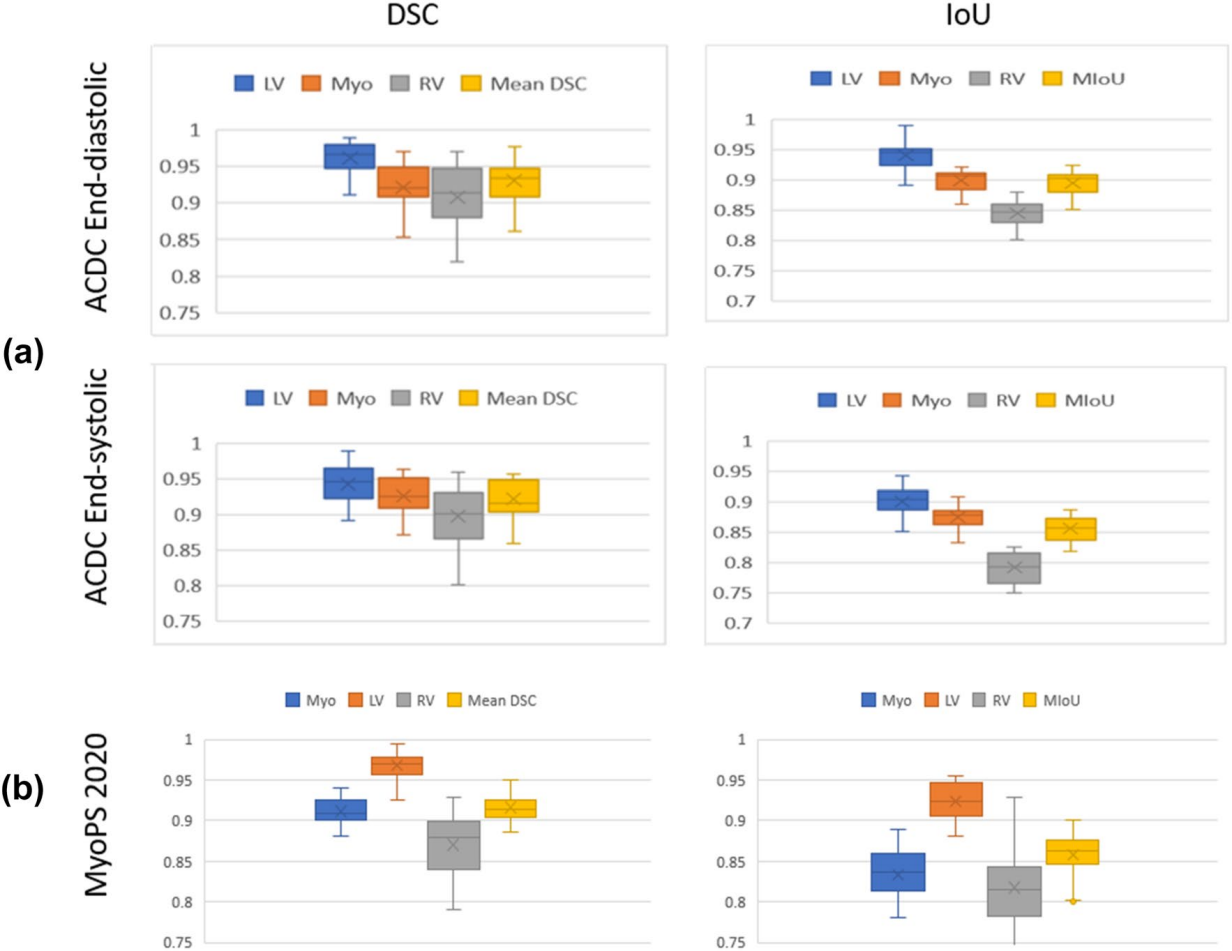
Box plots of the CAT-Seg framework results in terms of DSC and IoU **a** on ACDC dataset for the three cardiac substructures (LV, Myo, and RV) and the mean IoU and DSC in both cardiac phases (ED and ES), **b** on MyoPs 2020 dataset for external validation

Another aspect is investigated to show the stability in CAT-Seg performance, the mean and range of the results are shown by boxplot in Fig. 9. It demonstrates that the range of segmentation results in terms of both DSC and IoU is compact and consistent for all three substructures. In Fig. 9a, the segmentation results of ACDC 2017 are presented. The LV segmentation results show that the DSC results are symmetric in both cardiac cycles. Also, the LV segmentation results are symmetric in terms of IoU results in the ES phase, but it has negative skew in the ED phase. Moreover, for both cardiac phases, the myocardium shows positive skew in DSC results, but it has a negative skew in IoU results. Additionally, the RV shows a spread in both cardiac phases but most of the results are symmetric. It has segmentation results that are consistent in terms of IoU than DSC. It is notable that the results in all cases are consistent with no outliers shown. The Mean IoU result in the ED cardiac phase is 0.8946 ± 0.0190 and 0.8554 ± 0.0201 in the ES cardiac phase. In the ED cardiac phase, the mean DSC is 0.9298 ± 0.0270 and 0.9216 ± 0.0256 in ES cardiac phase. The shown results covey the stable performance of CAT-Seg with minimal fluctuation in performance. Moreover, the CAT-Seg is tested using an external test set from MyoPs 2020 dataset to show the robustness of the framework, the mean and range of the results are shown by boxplot in Fig. 9b. It demonstrates that the range of segmentation results in terms of both DSC and IoU is compact and consistent for all three substructures. It is notable that the results in all cases are consistent with no outliers shown. The LV DSC and IoU results are 0.967395 ± 0.015953 and 0.924215 ± 0.021997 respectively with a small standard deviation that doesn’t exceed 2.2%. Also, Myo segmentation has a small standard deviation account to 1.6156% in DSC measure and 3.0739% in the IoU measure with average DSC and IoU of 0.911325 and 0.832885 respectively. While RV has the highest standard deviation due to the variation between RV in the ACDC 2017 and the MyoPs 2020. The DSC and the IoU results for segmenting RV are 0.870285 ± 0.041033 and 0.817455 ± 0.055544 respectively.

**Fig. 10:**
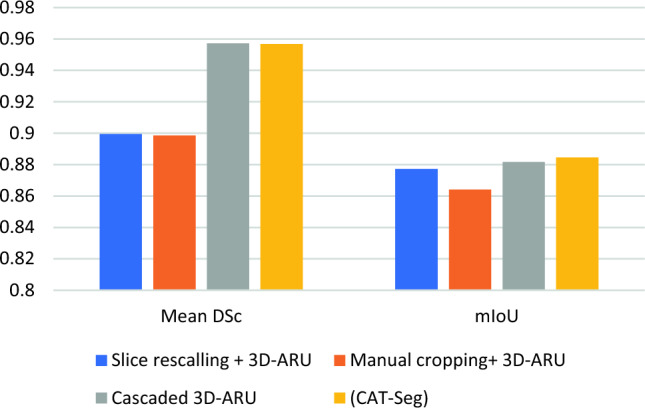
Mean DSC and mIoU on ACDC dataset comparison between the whole slice and the three types of localized slices

Figure 10 depicts the importance of the localization phase as it compares the using the 3D-ARU in segmenting different types of slices in terms of mean DSC and mIoU. First, it uses the full slice without any localization or annotation and thus it results in relatively low segmentation results due to the complex structure of the cardiac MRI and surrounding objects. Then, the manually cropped slices were extracted as 128*128 blocks taken from the center following the standard used in the literature [14], 16. These slices are input to the proposed 3D-ARU model, but it also reflects a low segmentation evaluation. Moreover, cascaded 3D-ARU and the proposed model compete in the evaluation of the segmentation as both show approximately the same results in terms of mean DSC and mIoU. However, the proposed model takes roughly less than half of the testing time of the cascaded 3D-ARU.

## Discussion

The performance of CAT-Seg is compared to existing approaches on the ACDC and MyoPs 2020 datasets for further validation. The comparison between the results for biventricular segmentation on ACDC dataset is shown in Table 6. CAT-Seg significantly outperformed all other methods in terms of the DSC and IoU on the ACDC test dataset. Since most of the state-of-the-art methods used DSC to evaluate the segmentation results, Table 6 details the evaluation comparison in terms of DSC. It is worth noting that the segmentation effect is particularly good for the more difficult segmentation of the ES of the heart. CAT-Seg is able to segment LV, Myo, and RV with an average minimum performance gap of 1.165%, 4.36% and 3.115% respectively. While the average maximum improvement in segmenting LV, Myo and RV is 4.395%, 6.84% and 7.315% respectively. The proposed model outperforms Li et al. [30] in LV, Myo and RV segmentation by 0.32%, 6.40%, and 1.15% respectively in ED cardiac phase. Also, in ES cardiac phase compared to Li et al. [30] the proposed model shows an outstanding performance in segmenting LV, Myo and RV by a performance gap of around 3.87%, 4.28%, and 5.08%. Furthermore, the proposed model is able to segment LV with a DSC that is 1.295% higher than that of the Yang et. al [13] work. Also, it is able to segment RV with a DSC that is 4.065% higher than that of the Yang et. al [13] model. Furthermore, the improvement in segmentation Myo is 4.36% in DSC compared to Yang et. al [13] model. Moreover, the CAT-Seg outperforms Silva et al. [32]’s model in segmenting the three substructures in both ED and ES phases. It is able to segment LV with a DSC that is 1.3% and 3.5% higher than that of the Silva et al. [32] model in the ED and ES phases. Also, the improvement in segmentation Myo in DSC is 6.38% for ED and 6.57% for ES compared to Silva et al. [32] model. Additionally, it is able to segment RV with a DSC that is 2.58% and 8.65% higher than that of the Silva et al. [32] model in the ED and ES phases respectively. Although the proposed model shows low average improvement in segmenting LV in ED, it draws an average improvement of 4.5316% in segmenting the three cardiac substructures in the ES cardiac phase. Moreover, the outstanding performance of the proposed model in segmenting Myo and RV in ES cardiac phase improvement in the ES phase. Additionally, it reflects the strength of the proposed model to solve the mentioned challenge of ES segmentation especially for RV.

**Table 6:** Comparison with state-of-the-art cardiac segmentation methods on ACDC dataset in segmenting (LV, Myo and RV) in terms of DSC for both cardiac phases

Author	Cardiac phase	DSC		
		LV	Myo	RV
Li et al. [30]	ED	0.9700	0.8900	0.9400
	ES	0.9300	0.9100	0.8900
Wu et al. [33]	ED	0.9593	0.8960	0.9133
	ES			
Yang et. al [13]	ED	0.9580	0.9098	0.9055
	ES			
Silva et. al [32]	ED	0.9602	0.8902	0.9257
	ES	0.9337	0.8871	0.8543
Sharan et.al [10]	ED	0.9210	×	×
	ES		×	×
Yang et al. [22]	ED	0.8982		
	ES			
Wang et al. [34]	ED	0.9620	×	×
	ES	0.9390	×	×
Budai et al. [20]	ED	0.9270	×	0.873
	ES		×	
Cheng et al. [17]	ED	0.9490	0.888	0.888
	ES			
Abdeltawab et al. [16]	ED	0.9600	0.8800	×
	ES	0.9200	0.8900	×
CAT-Seg	ED	0.9732	0.9540	0.9515
	ES	0.9687	0.9528	0.9408

The performance of CAT-Seg is compared to existing approaches on the MyoPs dataset for further validation. The comparison between the results for biventricular segmentation is shown in Table 7. CAT-Seg significantly outperformed all other methods in terms of the DSC on the MyoPs test dataset. CAT-Seg is able to segment LV, Myo, and RV with an average minimum performance gap of 6.13%, 5.44% and 2.912% respectively. While the average maximum improvement in segmenting LV, Myo and RV is 14.26%, 10.37%, and 8.544% respectively. It is worth emphasis that the results shown in Table 7 for CAT-Seg are without training on the training set of MyoPs 2020 and succeeded to surpass the performance of the state of the art. Hence, elucidate the generalization and robustness of the framework.

**Table 7:** Comparison with state-of-the-art cardiac segmentation methods on the MyoPs2020 dataset in segmenting (LV, Myo and RV) in terms of DSC

Author	Myo	LV	RV
Li et al. [35]	0.868	0.914	0.848
Zhao et al. [36]	0.847	0.849	
Li et al. [30]	0.8293 ± 0.1038	0.8600 ± 0.1057	0.804 ± 0.2696
CAT-Seg	0.9153 ± 0.0200	0.9701 ± 0.0191	0.8727 ± 0.0412

CAT-Seg attempts to provide a balance between the number of parameters and the accuracy, as the proposed SqueezeDeepLabv3+ uses SqueezeNet which is a lightweight and efficient CNN model. Also, it has fewer parameters than Xception so the SqueezeDeepLabv3+ decreases the number of parameters by 40.1173% and improves the accuracy by 1.3623% over the original DeepLabv3+. While the proposed 3D-ARU increases the number of parameters by 23.9719% over the original ResUNet but it improves the accuracy by 1.1615% compared to the original architecture. So, CAT-Seg framework compromises the number of parameters by using SqueezeNet for decreasing number of parameters and Attention mechanism which improves the accuracy, but it has greater number of parameters.

## Conclusion

In this study, a fully automatic multi-stage segmentation framework CAT-Seg is proposed. The proposed framework is composed of two proposed architectures. In the first, ROI is localized by the modified variant SqueezeDeepLabv3+, to minimize processing and address the issue of pixel class imbalance. The proposed architecture for SqueezeDeepLabv3+ uses SqueezeNet to enrich the encoder path. Also, SqueezeDeepLabv3+ modifies the atrous rate to localize the small structures like RV in ES. The second step involves submitting the ROI to 3D-ARU for segmentation. The proposed 3D-ARU uses ResUNet incorporating a spatial attention mechanism.

The results of the experiments show that the proposed method produces a mean DSC of 0.9595 in ED and 0.9541 in ES. In comparison to the single-stage segmentation process, the division into steps performed better. This is supported by the evaluation of the performance using the ACDC 2017 test dataset, where the proposed method achieves higher performance compared to state-of-the-art approaches in segmentation. CAT-Seg achieved an average maximum improvement in segmenting LV, Myo and RV of 4.395%, 6.84% and 7.315% respectively. Similar results are achieved when applied on the test set only of MyoPs 2020, producing a mean DSC of 0.9163 and mIoU of 0.8581. In conclusion, CAT-Seg offers a useful assistive tool to aid the early detection and treatment planning of cardiovascular diseases, which is critical for a better prognosis. For future work, this study can be extended and applied to 3D medical images augmentation, which can solve the limitation of limited dataset and reflect the changes in more samples.

## Data Availability

ACDC: Automated Cardiac Diagnostic Challenge Dataset from the 2017 MICCAI challenge. The ACDC dataset is available via http://humanheartproject.creatis.insalyon.fr/database/#collection/637218c173e9f0047faa00fb. MyoPs: Myocardial pathology segmentation combining multi-sequence CMR from the 2020 MICCAI challenge. The MyoPs dataset is available via https://mega.nz/folder/BRdnDISQ#FnCg9ykPlTWYe5hrRZxi-w
